# Recapitulation of Ayurveda constitution types by machine learning of phenotypic traits

**DOI:** 10.1371/journal.pone.0185380

**Published:** 2017-10-05

**Authors:** Pradeep Tiwari, Rintu Kutum, Tavpritesh Sethi, Ankita Shrivastava, Bhushan Girase, Shilpi Aggarwal, Rutuja Patil, Dhiraj Agarwal, Pramod Gautam, Anurag Agrawal, Debasis Dash, Saurabh Ghosh, Sanjay Juvekar, Mitali Mukerji, Bhavana Prasher

**Affiliations:** 1 Genomics and Molecular Medicine, CSIR-Institute of Genomics and Integrative Biology, New Delhi, India; 2 CSIR’s Ayurgenomics Unit–TRISUTRA (Translational Research and Innovative Science ThRough Ayurgenomics) CSIR-Institute of Genomics and Integrative Biology, New Delhi, India; 3 Academy of Scientific and Innovative Research (AcSIR), CSIR-IGIB, Delhi, India; 4 G.N.Ramachandran Knowledge Centre for Genome Informatics, CSIR-Institute of Genomics and Integrative Biology, New Delhi, India; 5 Vadu Rural Health Program, KEM Hospital Research Centre, Pune, India; 6 Human Genetics Unit, Indian Statistical Institute, Kolkata, India; Estonian Biocentre, ESTONIA

## Abstract

In Ayurveda system of medicine individuals are classified into seven constitution types, “*Prakriti*”, for assessing disease susceptibility and drug responsiveness. *Prakriti* evaluation involves clinical examination including questions about physiological and behavioural traits. A need was felt to develop models for accurately predicting *Prakriti* classes that have been shown to exhibit molecular differences. The present study was carried out on data of phenotypic attributes in 147 healthy individuals of three extreme *Prakriti types*, from a genetically homogeneous population of Western India. Unsupervised and supervised machine learning approaches were used to infer inherent structure of the data, and for feature selection and building classification models for *Prakriti* respectively. These models were validated in a North Indian population. Unsupervised clustering led to emergence of three natural clusters corresponding to three extreme *Prakriti* classes. The supervised modelling approaches could classify individuals, with distinct *Prakriti* types, in the training and validation sets. This study is the first to demonstrate that *Prakriti* types are distinct verifiable clusters within a multidimensional space of multiple interrelated phenotypic traits. It also provides a computational framework for predicting *Prakriti* classes from phenotypic attributes. This approach may be useful in precision medicine for stratification of endophenotypes in healthy and diseased populations.

## Introduction

In the present era of phenomics, there has been an increase in emphasis on endo-phenotyping along with omics approaches for identification of groups that differ in susceptibility, prognosis and therapeutic requirements [1,2]. This has formed the basis for the development of predictive preventive, personalised and participatory (P4) medicine [3–5]. There is an unmet need for the development of adequate phenotyping methods for stratification of healthy individuals at a systemic level. Although methods/questionnaires are available for classification of individuals in terms of specific anatomical and physiological attributes such as somatotypes, phototypes, chronotypes and metabotypes [6–8]. These have been to some extent useful in predicting the health and disease tendencies with respect to specific systems.

Phenotypic stratification of healthy individuals forms the primary basis for predictive and personalised medicine in Ayurveda, a 5000 year old Indian system of medicine [9,10]. According to this system, individuals are classified into seven broad constitution types “*Prakriti*” that is determined at the time of birth and remains invariant throughout life. Amongst the seven types, three groups *Vata* (V), *Pitta* (P) and *Kapha* (K) are at the end of the phenotypic spectrum and are described to have contrasting disease susceptibilities [11]. V, P and K are herein referred to as extreme (distinct) *Prakriti* while other four *Prakriti* types VP, PK, VK and VPK are referred here as non-extreme *Prakriti*. An earlier study has revealed molecular differences between the extreme *Prakriti* types [12]. Assuming that the *Prakriti* types correspond to objectively identifiable sub-phenotypes, they should form clusters within a multidimensional space where the axes conform to the phenotypic traits used for clinical stratification. This would be most obvious for extreme *Prakriti*s and should conceptually be extendable to non- extreme *Prakriti*s. To optimally extract useful knowledge contained within Ayurveda, it is important to develop a modern framework in which such assumptions are tested, followed by development of methods that would enable standardized implementations that are rapid, accurate and scalable[12–14].

**Fig 1:**
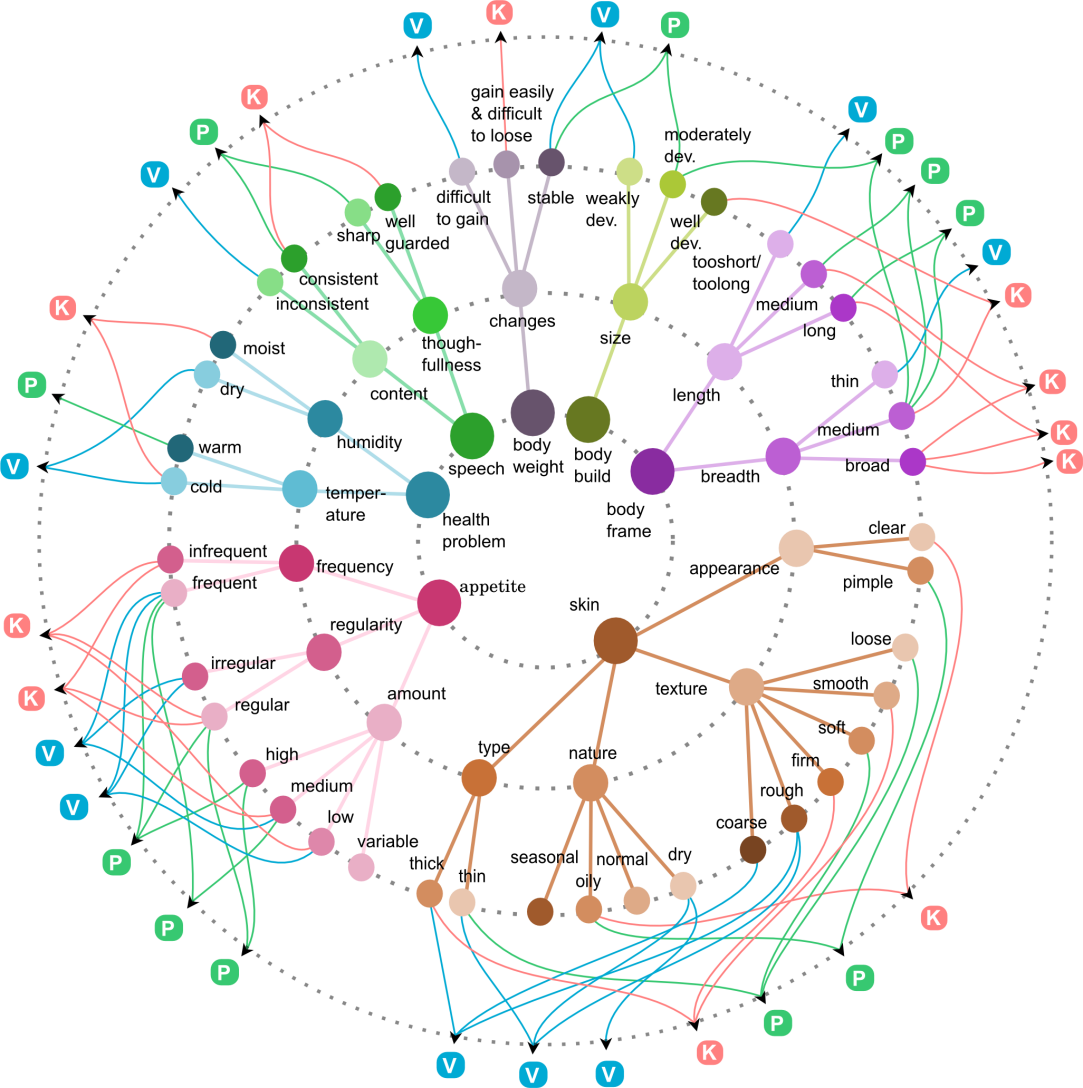
Visual representation of *Prakriti* interpretation process based on original textual references. *Prakriti* interpretation is based on the combinatorial occurrence of phenotypic feature that are captured through the questionnaire. This is an illustration with few examples of features. The three inner concentric circles represent feature category; feature sub-class and feature values of the questionnaire in each of the sub-classes. The outermost circle indicates the final interpretation in terms of *Vata* (V)/ *Pitta* (P)/ *Kapha* (K) based on the different combinations of values. For example, if skin type is thin it could be either due to *Vata* or *Pitta* however if it also dry and rough it would be interpreted as *Vata* type whereas if it is oily and loose or soft it would be considered as *Pitta* type. Similarly, if someone has a health problem in cold it could be *Vata* or *Kapha* type but humidity can further segregate it viz. health problem in moist with cold is for *Kapha* type whereas problem in cold and dry would be observed in *Vata* type.

*Prakriti* assessment involves examination of more than 150 features with anatomical and physical activity related attributes that can be directly examined and physiological and psychological parameters inferred based on the responses of the individual and past history[13]. During the examination, care is taken to avoid misinterpretation of the clinical features that might arise due to recent or occasional change of internal or external environment. The process of inferring *Prakriti* from these large number of features is a non-trivial task. Cumulative assignment to a *Prakriti* group involves consideration of individual features as V, P or K. However, in majority of instances this involves assignment based on the combinatorial occurrence of features (**Fig 1**). Although this is a system standardized with guidelines from well documented Ayurveda texts, which also takes the interactions into account, the decision rules have not yet been formalized as mathematical models. One of the major challenges in building these models is the small ‘n’ and large ‘p’ problem where ‘n’ refers to number of samples and ‘p’ refers to no of parameters. This type of data matrix results in multicollinearity that arises when many features are correlated amongst each other [15,16]. In order to address this problem, we have used advanced modelling approaches of LASSO [17], elastic net [18] and random forests [19]. We have also used unsupervised approach in a purely data driven fashion [20–22]. Unsupervised clustering provided three natural clusters that corroborate with the clinicians’ classification of V, P and K. Supervised modelling allowed us to identify core set of variables that were concordant between all the three methods and could accurately predict extreme *Prakriti* with high specificity and sensitivity. This is the first study of its kind that not only highlights the specific attributes that help in distinguishing constitution types amongst healthy individuals in an unbiased approach but also recapitulates the connectivity between seemingly unrelated systems that are described in Ayurveda.

## Methods

The study was carried out as per protocols approved by the institutional ethics committee at CSIR-Institute of Genomics and Integrative Biology, Delhi and KEM Hospital Research Centre, Pune, India. Recruitment of volunteers and sample collection was carried out using standard procedures following ethical guidelines of Indian Council of Medical Research, India for biomedical research and informed consent of volunteers.

### Recruitment of subjects and clinical assessment of *Prakriti*

The study was carried out in a genetically homogeneous rural cohort developed under Vadu Rural Health Program (VRHP) for Health and Demographic Surveillance System (HDSS) near Pune in the western part of India. This cohort is henceforth referred to as Vadu cohort. 10,100 individuals of equal number of healthy males and females between the age groups of 18–40 years residing in 22 villages of VRHP area were pre-screened by field research assistants for identification of prospective subjects of extreme *Prakriti*. Detailed *Prakriti* evaluation was carried out in 528 individuals by Ayurveda physicians using the questionnaire that had been developed in the earlier study in the North Indian population [12]. In order to have homogeneity in administering the questionnaire an extensive training was provided to all the Ayurveda clinicians. In addition, a manual was developed to enable clinical decision-making. Based on the physicians’ judgment and the responses of the subjects the individuals were classified into one of the seven sub-types; *Vata* (V), *Pitta* (P), *Kapha* (K), VP, VK, PK and VPK. Assignment to *Prakriti* groups was done by two groups of Ayurveda physicians, one at the field site where the clinician who carried out the *Prakriti* examination at site and the second group of physicians at CSIR-IGIB including Ayurveda co-investigator who assigned *Prakriti* based on the data collected at the field site. A subset of study subjects were examined by both groups of physicians for cross validation of observation and questionnaire data. There was a cross validation exercise of nearly 20% of the subjects by different Ayurveda clinicians.

### Preprocessing of data

Collected questionnaire data is stored in a database. Data were pre-processed, variables with more than 5% missing values were removed while others were imputed with the mode of the data. Non-varying factor variables were then excluded from further analysis. 133 features/questions were included for the final study.

We followed both unsupervised and supervised machine learning approaches using phenotype data from two cohorts from different geographical locations. A flowchart describing the steps followed have been provided as supplementary figure **(S1 Fig)**

### Genetic homogeneity of the population

Genotype data was generated on 237 samples of different *Prakriti* types from the Vadu cohort, on Affymetrix Genome Wide Human SNP array 6.0 (Affymetrix, Santaclara, CA, USA). The genetic relatedness and homogeneity of the VADU cohort with Indian population was established by analysis of the VADU genotype data with shared SNPS in the Indian Genome Variation Consortium panel. The IGVC database houses genotype data of representative samples from genetically and ethnically diverse populations of the country [23]. A set of 17,675 SNPs that were shared with 509 Indian Genome Variation Consortium (IGVC) samples generated from Affymetrix 50k Xba1 240 Gene chip Human mapping array were used for the PCA analysis. All the SNPs used for the analysis followed Hardy-Weinberg equilibrium and qualified all the standard quality criteria such as more than 90% genotyping call in more than 90% of samples. We ensured that all the genotype data were from the same strand prior to merging the data. Principal Component analysis (PCA) of the genotype data was performed using EIGENSOFT 5.0 [24,25]

### Unsupervised clustering of individuals

Clustering analysis based upon questionnaire features was applied to discover the inherent structure and to stratify individual subjects. A random forests model consisting of 1 million decision trees was constructed in unsupervised mode to derive similarity (1-dissimilarity) matrix. This matrix further was used for objectively evaluating optimum cluster number through partition around medoids (PAM) [26,27] with cluster numbers varying from 2 to 20. We used silhouette width as a criterion to identify optimum cluster numbers [28]. A visual inspection of the clusters was done using Multi-Dimensional Scaling plot. Thereafter robustness of clustering was assessed through a permutation of original features. One hundred times permuted datasets were generated and for each permutation, similarity matrix was created with one million decision trees using random forests algorithm. Silhouette widths derived from permuted datasets were plotted vis-a-vis original data through a visualization approach, we called as Savannah plot. Permutation analysis also helped us to test if there exists any coupling between the features of the questionnaire data with respect to *Prakriti* groups. A similar analysis was carried out on all samples to test whether non-extreme samples were indeed a mixture of the constituent *Prakriti* types. Whole analysis was implemented in R statistical programming language [29] using randomForest [30] and cluster package [31].

### Supervised modeling of the questionnaire

Three methods were used for the modelling of the questionnaire. A brief description highlighting the need for adopting these advanced methods is provided below.

#### LASSO model

The regression framework of the LASSO model was used for extreme *Prakriti* modelling as it addresses the problems of multicollinearity arising out of large number of explanatory variables. It includes a penalty function in the model that shrinks the regression coefficients of insignificant or unnecessary explanatory variables to zero [17,32]. The model is given by,

$$Y=X\beta +\lambda \;\left|\beta_{j}\right|+\varepsilon$$

Where Y is the vector of responses, X is the design matrix comprising of the explanatory variables, β = (β_1_, β_2_ … β_j_) is the vector of regression coefficients, λ is a tuning parameter that controls model accuracy and ε is the random error component. In our study, vector Y is the *Prakriti* label V/P/K assigned to the subjects X is the matrix containing feature values collected through the questionnaire.

#### Elastic net model

When the data have highly correlated predictors LASSO tends to select only one variable and removes the correlated variable/s. Since there might be some interaction involved in the variables, grouped selection might be needed for future reference which is done in elastic net. In our study, we anticipate that non-redundant variables alone might not differentiate extreme from non-extreme *Prakriti* individuals and retaining correlated variables might be of significant importance. Hence, we have also used elastic net method. Elastic net regression is related to LASSO in the sense that it also uses penalty parameter to circumvent the problem of multicollinearity and belongs to penalized regression family. It is given by,

$$Y=X\beta +\lambda \left[\left(1-\alpha \right)+∥\beta {∥}_{2}^{2}/2+\alpha ∥\beta {∥}_{1}\right]$$

Elastic net method is governed by two parameters α and λ. Lambda (λ) is same as in LASSO regression while alpha (α) should strictly be between 0 and 1 for elastic net [18].

#### Random forests model

Random forests (RF) is an ensemble decision tree based algorithm, where each decision tree is built independently from different bagging samples and randomly selecting a subset of features (square root of total number of variables for classification task). Two parameters namely ntree (number of decision trees) and mtry (a subset of features to be chosen randomly) were optimized before building the final training model [19]. Variable selection was performed using Boruta package in R [33]

#### Modelling strategy

**Fig 2:**
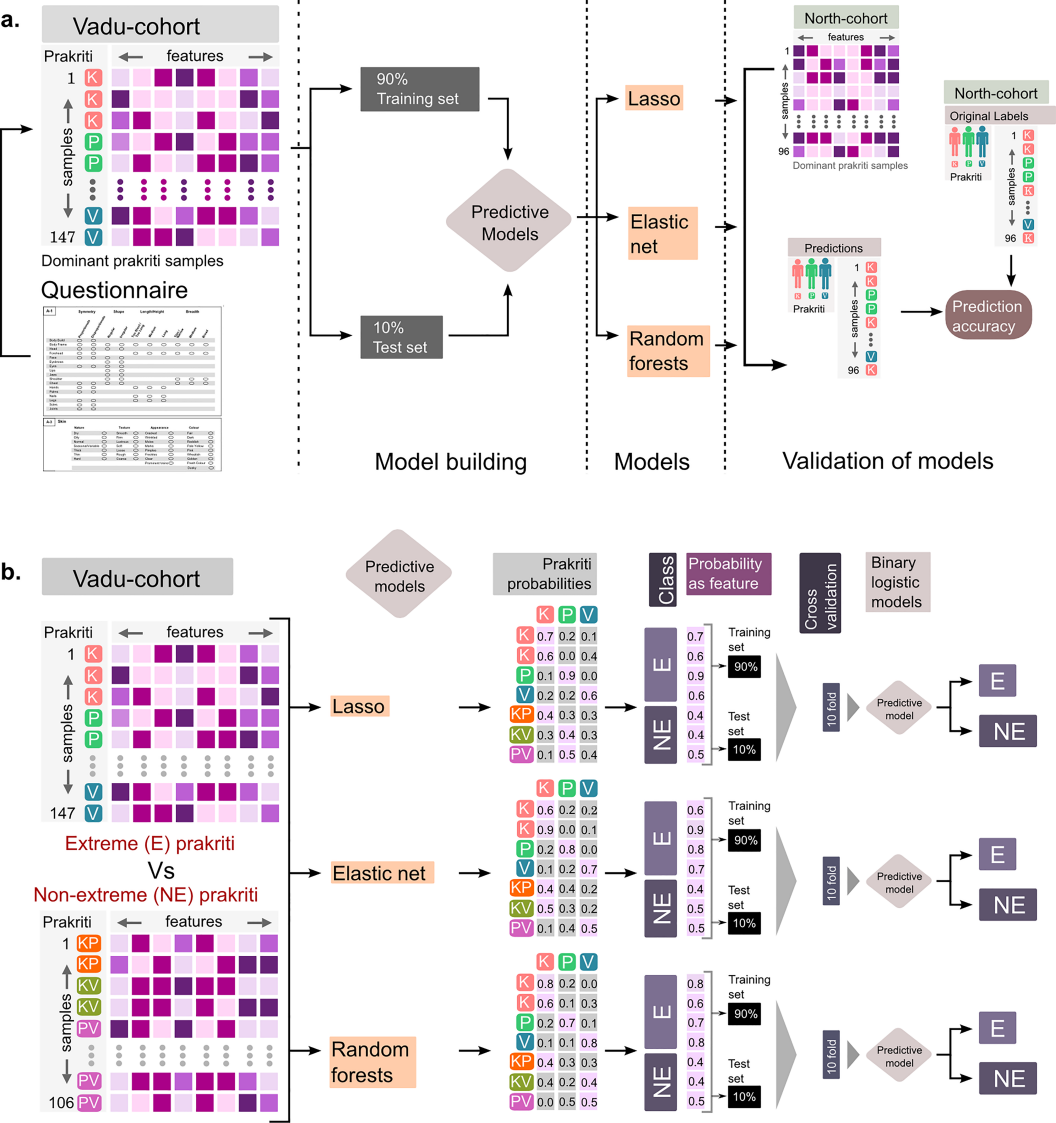
Schematics demonstrating modelling strategy. Schematic showing the approach taken for modelling of (A) extreme *Prakriti* types followed by modelling of (B) extreme vs non-extreme using probability score generated from extreme *Prakriti* model for all the three methods. Maxima Probability scores were utilized to create binomial logistic regression for classification of extreme vs non-extreme.

**Extreme*Prakriti* modelling.** Data partition was fixed for all downstream modeling strategies. From the extreme set of individuals (total = 147; V = 66, P = 35, K = 46), a random sample of 90% data was drawn to create a training set. The remaining 10% data was used for testing the models [**Fig 2**]. Out of 147 extreme *Prakriti* samples 81 samples were females (23 *Kapha*, 14 *Pitta*, 44 *Vata*) and 66 samples (23 *Kapha*, 21 *Pitta* and 22 *Vata*) were males.

A battery of LASSO, elastic net and random forests was then applied for supervised classification modeling.

Features for LASSO and elastic net models were selected by the optimization of penalty parameters [λ], [λ, α] respectively through 10 fold cross-validation. In the case of elastic net a two dimensional cross validation was performed for the tuning of parameters since it involves two parameters. A grid of values (0 to 1, separated by 0.1) was picked for α. Then for each alpha a cross validation was performed to select other parameter λ. Minimum λ was chosen based on the model error. So for each “α” a λ value and corresponding model accuracy was obtained. Finally, optimum α was chosen based on the minimum model error.

For random forests, feature selection was done using the Boruta algorithm [33] followed by optimization of number of trees and number of variables (ntree and mtry respectively). Twenty models each with ntree ranging from 500 to 10000 (step-size = 500 trees) were built and least median Out of Box (OOB) in conjunction with least standard deviation criterion were used to finalize the optimal ntree as 4000. A similar strategy was followed to derive the optimal mtry as 5. All tuning and optimization were implemented in R statistical language using glmnet [34] package for LASSO and elastic net and randomForest package [30] for RF.

### Validation of models on an external dataset from a different population

The models were validated in a dataset of an earlier study from the North Indian population. Since 106 features were common between both the datasets after pre-processing we rebuilt the model using the same methods on the VADU data. A similar strategy of 90% data for training and 10% VADU data was used as test set. The model so built on VADU cohort was then tested on the complete data of 96 subjects (48 subjects each from males and females with nearly equal representation of *Kapha*, *Pitta* and *Vata*) from the North Indian cohort (**Fig 2**).

### Model for segregation of Extreme vs Non-Extreme *Prakriti*

In order to segregate extreme from non-extreme samples in a heterogeneous population, we built a model with a hybrid approach. In this approach, the extreme *Prakriti* model was run on a mix of 147 extreme and 106 non-extreme samples and maxima of membership-probability were recorded. We expected that score for each *Prakriti* generated based on the above model would assign a high probability to the extreme samples in one class whereas the non-extreme would not show such skewness towards one group. These scores were then used to construct the extreme versus non-extreme models through binary logistic regression. Subsequently, tenfold cross validation was performed to test the robustness of these models (**Fig 2)**. Predictive performance of these models was then assessed on the left out set by calculating sensitivity, specificity from a confusion matrix [35] and the AUC of the ROC curve [36,37]

## Results

### Genotyping analysis confirmed the genetic homogeneity of the study population

Vadu cohort belongs to an Indo-European background from Western India. The genetic homogeneity of the cohort was confirmed using a panel of markers from Indian Genome Variation database (IGVdb) wherein the study population was found to be clustered with western populations from the IGVdb study. In general Vadu population is genetically homogenous, however few of the members appear as outliers in PCA plot (S2 Fig).

### Identification of extreme *Prakriti*

Since the questionnaire has three aspects including visual and tactile examination and subject's response, there could be inter-individual variability in administering the questionnaire as well as interpretation of the response. Assignment to *Prakriti* groups was done by two groups of Ayurveda physicians, one at the field site by the clinician who carried out the *Prakriti* examination and the second group who assigned *Prakriti* based on the data collected at field site. A total of 147 extreme *Prakriti* individuals were identified which consisted of three types namely *Kapha* (n = 46), *Pitta* (n = 35) and *Vata* (n = 66). Apart from extreme *Prakriti* type, 106 samples were also classified as non-extreme type VP, PK and VK.

### Emergence of three distinct robust clusters through unsupervised learning of questionnaire data from extreme *Prakriti*

**Fig 3:**
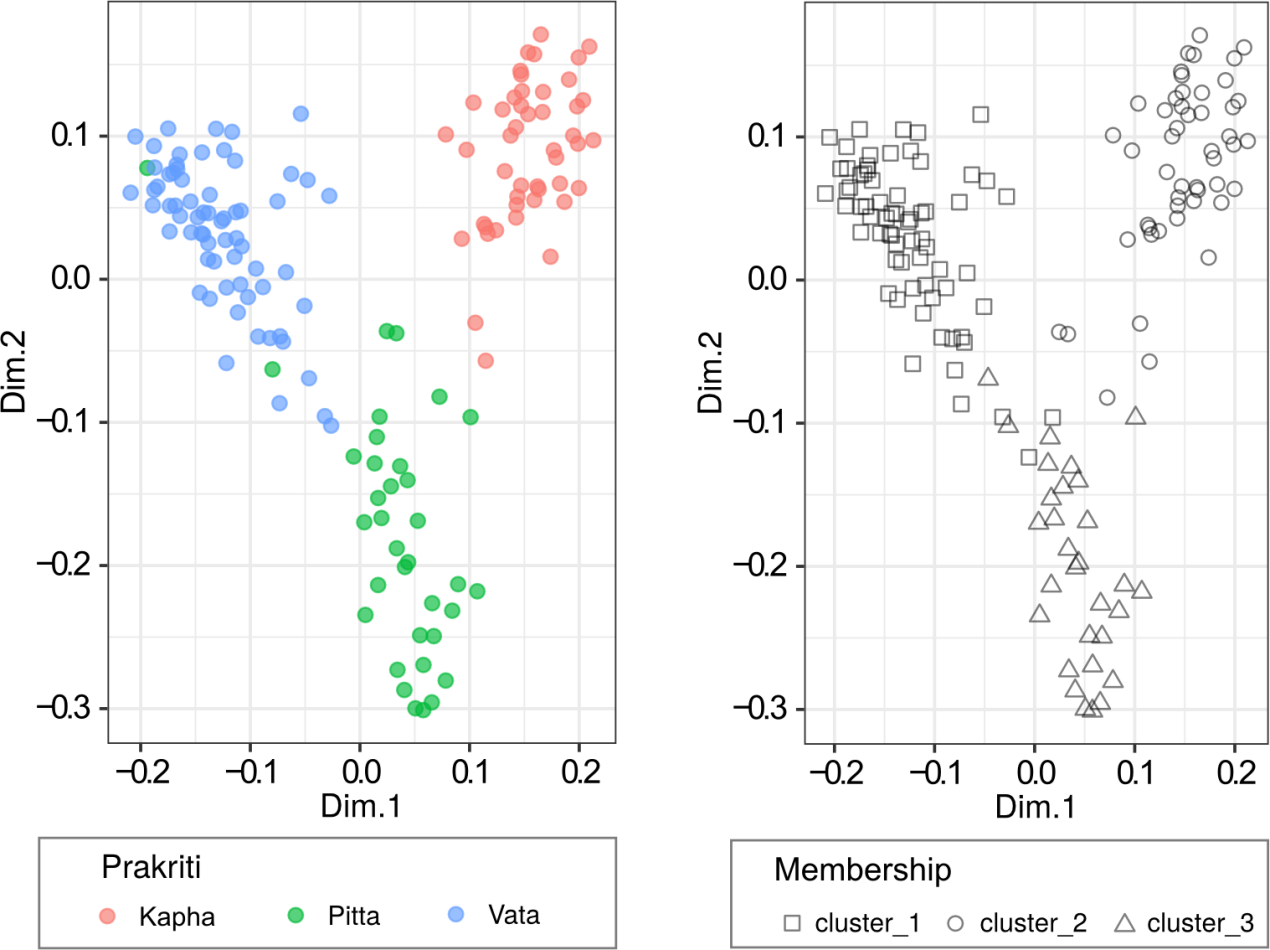
MDS Visualization for unsupervised clusters and original *Prakriti* labels from extreme *Prakriti* questionnaire data (male, female combined). Each shape refers to one individual. Unsupervised clustering of questionnaire data for extreme *Prakriti* individual gives rise to three clusters. These clusters correspond to original *Prakriti* membership as shown in the figure in the right panel.

**Fig 4:**
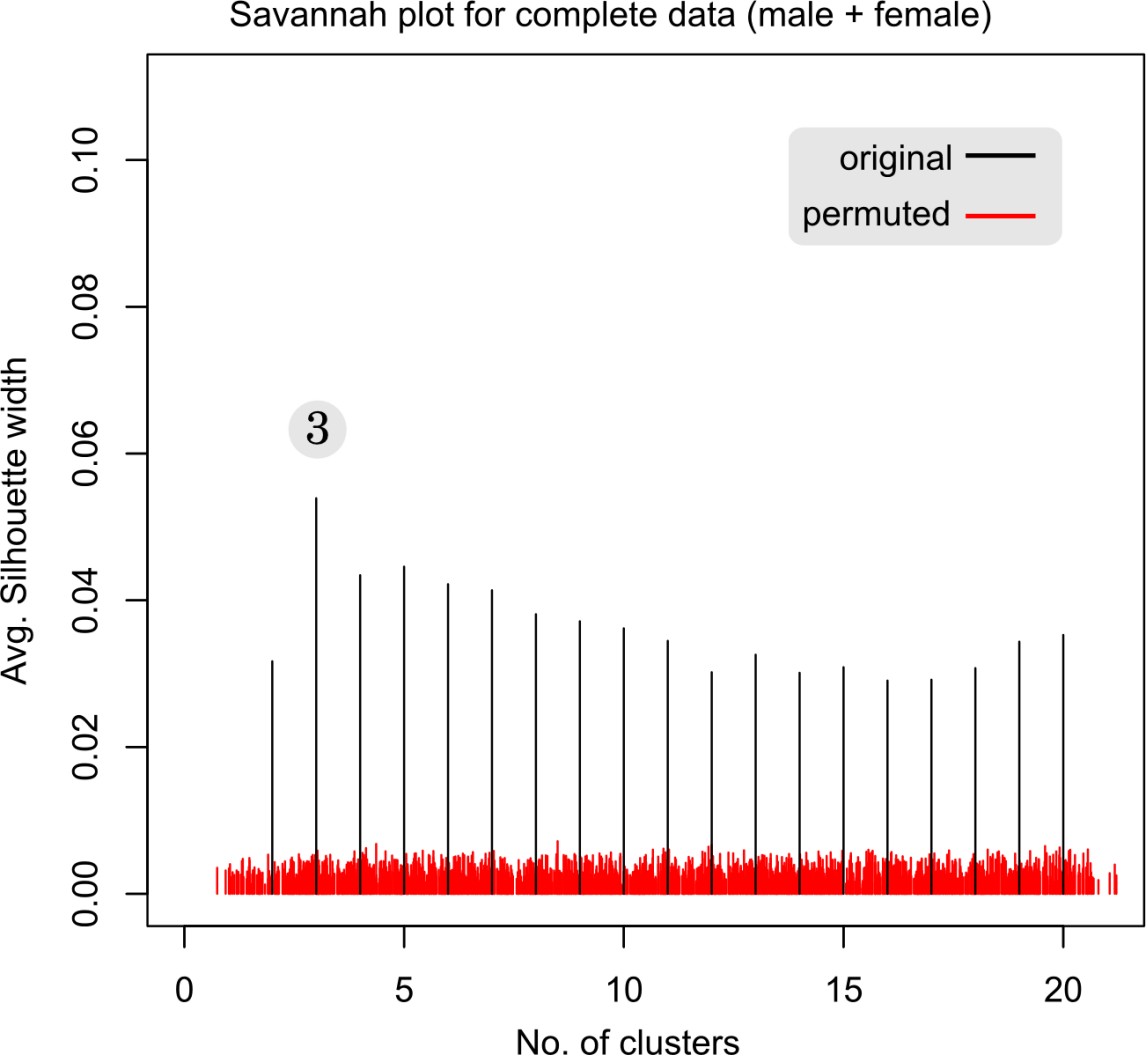
Savannah plot for extreme *Prakriti* questionnaire data (male, female combined) data. Black vertical lines represents average silhouette width for a given cluster number obtained from original data, based on which three clusters were found to be optimum. Red vertical lines in the background represent average silhouette width obtained from 100 permuted data. Average silhouette width from permuted data are smaller compared to original data and reveals robust nature of the cluster number derived from original data.

Unsupervised random forests based clustering revealed three clusters using purely a data-driven approach (**Fig 3**). These are evident in the Savannah plot in **Fig 4**. The line with the highest silhouette criteria represents the optimum number of clusters. Cluster memberships had 93.9% agreement with *Prakriti* labels thus proving the validity of *Prakriti* measurement through the questionnaire. Permutation analysis with the random shuffling of the feature values in the questionnaire data could not resolve into three clusters. The silhouette width for permuted data was observed to be manifold less in magnitude than those of the original data and was nearly uniformly distributed (**Fig 4**).

Similarly, unsupervised clustering of heterogeneous subjects revealed that non-extreme (VP,PK and VK) subjects occupied the intermediate space of the respective extreme *Prakriti*s and do not blend with the third extreme group that is not represented in them. For instance, KP *Prakriti* subjects map between the clusters occupied by extreme K and P subjects (**S3 Fig**).

Unsupervised clustering using random forests was also performed on male and female subjects separately. Three clusters were observed in males with 97% agreement (**Panel a in S4 Fig**). However, in the case of females four clusters were observed (Panel b in **S4 Fig**). This was also confirmed from the Silhouette width in the Savannah plot (**S5 Fig**). Though in the case of female data unsupervised clustering gives four clusters, two clusters were observed to be very close to each other. These close clusters correspond to *Kapha Prakriti* group.

This exercise revealed that there is a structure in the questionnaire data that led to the emergence of three clusters. The overlay of members within each cluster with a particular *Prakriti* group further substantiated the phenotype-to-phenotype linkages that led to clustering of samples within *Prakriti* types.

### Three distinct supervised learning approaches provide a core set of variables that accurately predict *Prakriti*

Unsupervised clustering using extreme subjects provided three distinct clusters. To identify a minimal set of variables as well as to capture the relationship in a mathematical manner between *Prakriti* types and feature attributes, we carried out supervised modelling using LASSO, elastic net and random forests algorithms.

**Fig 5:**
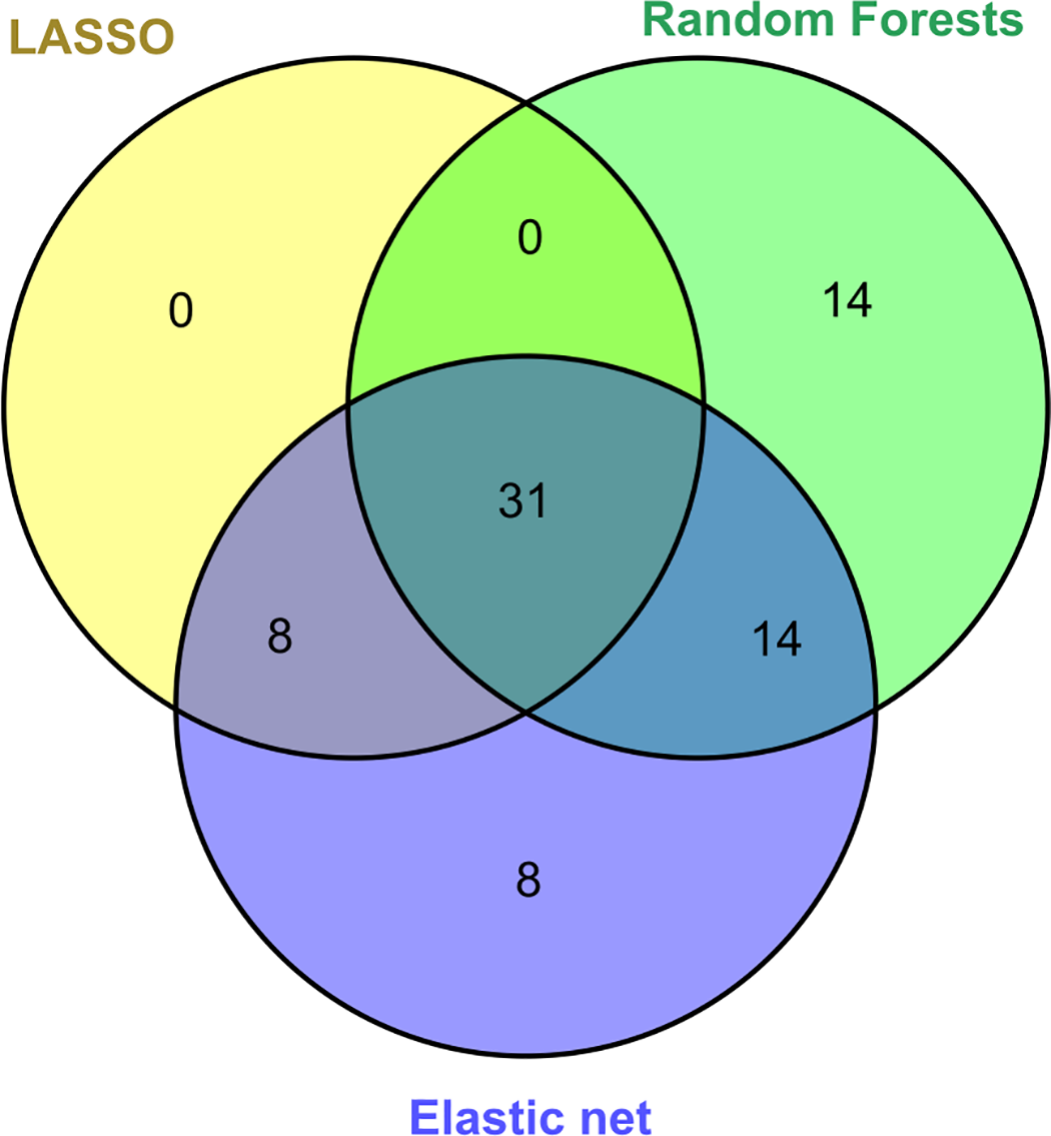
Feature Selection. Venn diagram shows the overlap of features selected through LASSO, elastic net and random forests modelling. 31 variables were common between all the three methods.

Through feature selection methods, we identified a minimal set of features from the 133 attributes that could most accurately identify the extreme groups from a heterogeneous population. We obtained 39, 61 and 59 features from LASSO, elastic net and random forests respectively (**S6, S7 and S8 Figs**). 31 features overlap in all the three methods. There were some features unique to random forests and elastic net (**S1 Table**). As anticipated, features from LASSO were a complete subset of elastic net (**Fig 5**). All the three models were tested using the 10% left out data set consisting of five samples of *Kapha*, 4 samples of *Pitta* and 7 samples of *Vata* types from the discovery cohort. As can be seen from the confusion matrices from the three algorithms, all the three models classify samples with 100% accuracy and perform equally on 10% hold-out set (**S2 Table**).

### Replication of *Prakriti* models across two cohorts of diverse genetic backgrounds

We wanted to test if predictive model built on one cohort would accurately predict *Prakriti* across different cohorts. We tested all the three models built on Vadu cohort in a North Indian data from our earlier study. The genetic homogeneity of the North Indian cohort was already established in earlier studies. The class-wise accuracy (sensitivity) for *Kapha*, *Pitta* and *Vata* was 93.1%, 82.7% and 94.7% respectively from the LASSO model and 96.5%, 86.2% and 97.3% respectively from elastic net (**Table 1**). The accuracy from random forests was 100% for *Kapha* and 79.3% for *Pitta* and 97.37% for *Vata*. The specificity from all the models were more than 90% (**Table 1**). Sensitivity and specificity were derived from confusion matrices of the respective models (**S3 Table).** There was a considerable overlap in the core set of variables identified from both models built on 133 and 106 features with LASSO (82%), elastic net method (94%) and random forests (91.3%). We also observed concordant results when we did a reverse procedure wherein the model built on North Indian data was tested on VADU data (**S1 Appendix**).

**Table 1:** Model summary for validation of North India data. *Prakriti* wise sensitivity and specificity of three models, LASSO, Elastic net and Random forests for validation of North India data.

	Sensitivity (%)			Specificity (%)		
	LASSO	Elastic net	Random forests	LASSO	Elastic Net	Random forests
***Kapha***	93.1	96.55	100	100	100	98.51
***Pitta***	82.75	86.2	79.31	94.02	97.01	98.51
***Vata***	94.73	97.36	97.37	91.37	93.1	91.38

### Development of a classifier for identification of extreme *Prakriti* groups from heterogeneous populations

**Fig 6:**
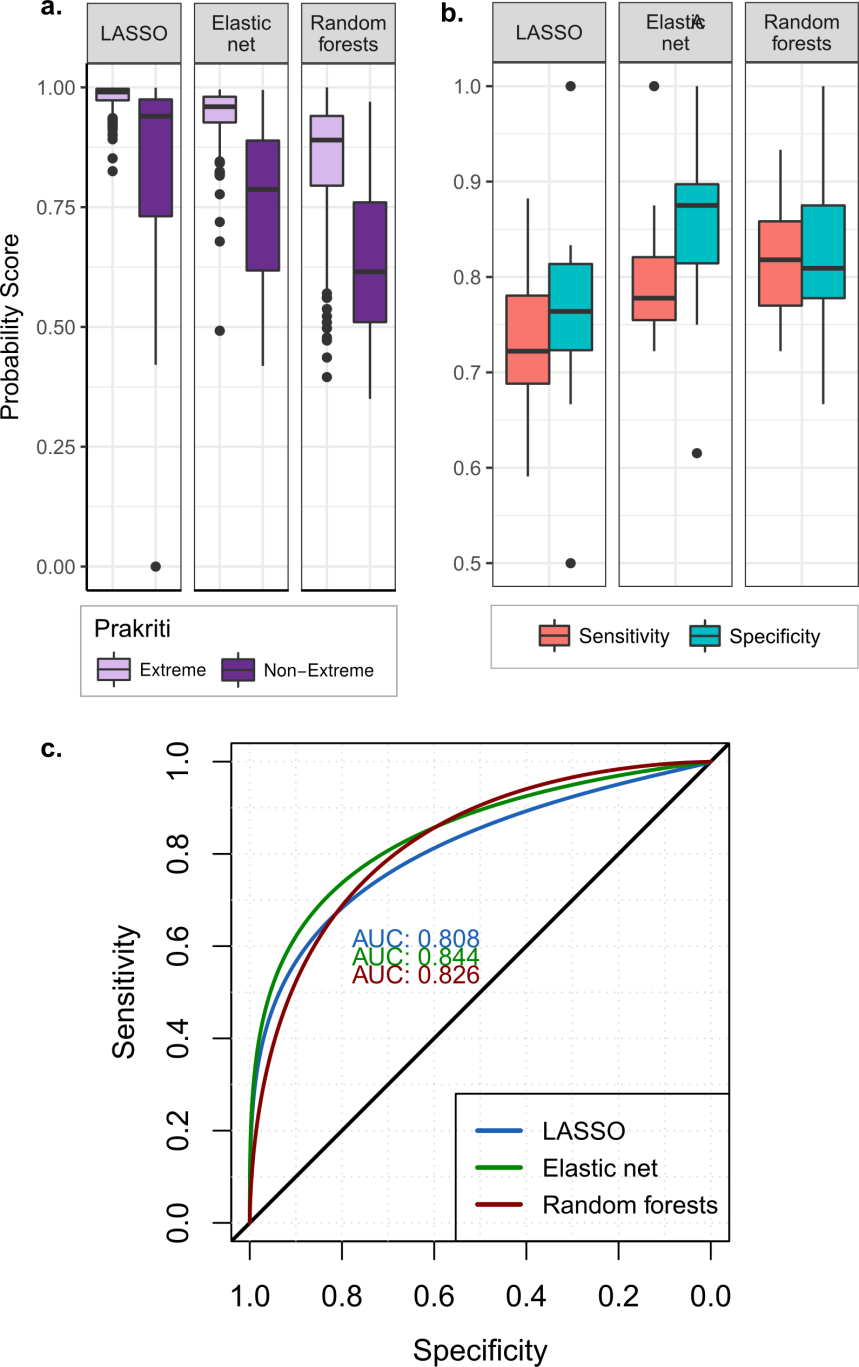
a) Boxplot for maxima of probability scores generated from extreme *Prakriti* models. Using extreme *Prakriti* models from all the three approaches probability scores were generated for all the samples. For extreme *Prakriti* the probability was high while for non-extreme *Prakriti* probability was comparatively less. The difference in distribution of probability score provided the basis for extreme vs non-extreme *Prakriti* modelling b) Boxplot of 10 fold cross validation for extreme vs non-extreme modelling. Using maxima probability score glm models were built to classify extreme from non-extreme *Prakriti*. 10 fold cross validation of the models shows good classification performance of models. Best performing models, one each from LASSO, elastic net and random forests were selected. c) ROC curve for distinguishing extreme from non-extreme *Prakriti*. Three best glm models selected each from LASSO, elastic net and random forests show good discriminatory ability as evident from AUC.

We wanted to test that if we provide a heterogeneous set of samples, do the methods identify extreme or non- extreme *Prakriti* with equal accuracy. Using the above model, probability distributions of *Prakriti*-membership scores upon non-extreme data did not show a preferential skew towards any of the labels (**Fig 6A**).

This finding was utilized to construct binary logistic regression (glm) upon the assigned probabilities to calculate the threshold for extreme versus non-extreme probability maxima. 10 fold cross-validation confirmed the robustness of the models (boxplot of model accuracy, **Fig 6B**) models built for classifying extreme classes from non-extreme classes.

Best models selected showed high sensitivity and specificity (>90%) (**Table 2).** Accuracy of the models were derived from the confusion matrices **(S4 Table**). ROC curve generated for best glm models made from probability score generated using LASSO, elastic net and random forests models showed considerable accuracy (>80%) as given by AUC (**Fig 6C**).

**Table 2:** Summary of models (extreme vs non-extreme modelling). Sensitivity and specificity of glm models built from probability scores obtained from LASSO, Elastic-net and Random forests model. The table shows the sensitivity and specificity for the best model each selected from three algorithms.

	Sensitivity (%)	Specificity (%)
**LASSO**	88	100
**Elastic net**	100	91
**Random forests**	93	90

## Discussion

Identification of target population for prediction and early actionable points of therapeutic intervention is the mainstay of precision medicine. The success of predictive marker discovery relies on homogeneity and endo-phenotyping of the study populations. Recently there has been an emphasis on studies involving extreme phenotypes for identification of at risk or protective markers as well as modifiers of diseases, differential drug requirements and dose response.

According to Ayurveda, individuals of different constitution types (*Prakriti*) have defined health and disease trajectories which encompass susceptibility to diseases, their prognosis as well as the suitability of diet and life style and responsiveness to drug and environment.

Study on extreme constitution types of Ayurveda which comprises nearly 10% of the population have revealed significant differences at the biochemical, expression, genetic level amongst the different constitution types [12,38,39]. Identifying predictive markers and modifiers relevant to high altitude adaptation and susceptibility to HAPE has also been demonstrated using these extreme phenotypes [40,41]. The potential of integration of this phenotypic classification method in conditioning complex genetic studies for addressing missing heritability [42] is also demonstrated.

Accurate identification of *Prakriti* relies on two major steps; (1) capturing a large number of multisystem phenotypes using a clinical questionnaire through history, examination and responses from the study individuals and (2) interpretation of the captured data taking into account all the inter-connectedness amongst sub-phenotypes with respect to different *Prakriti*. Cultural, regional as well as ethnic factors are also of considerable significance. The inter-observer bias as well as experience, observation and querying skills of the investigator administering the questionnaire are also critical. There is a need to minimise the number of variables without compromising on *Prakriti* prediction.

At the end of the study, we were able to set up a computational system to classify healthy individuals into different *Prakriti* types. We have demonstrated that phenotype data gives rise to three distinct clusters, which matches with the extreme *Prakriti* groups as classified by clinicians. We have also visualized intermediate *Prakriti* type and found that intermediate *Prakriti* is a mixture of constituent extreme *Prakriti* and does not blend with third *Prakriti* group. However, we also observed some overlap in extreme and non-extreme *Prakriti* group and we need to adopt or device methods for better resolution of extreme and non-extreme *Prakriti* separation. This was also reflected in extreme vs non-extreme *Prakriti* modelling where accuracy was reduced due to misclassification of non-extreme *Prakriti* type into extreme and vice versa. We have addressed this limitation using an indirect approach which uses extreme *Prakriti* model to generate probability score for all samples (extreme and non-extreme), subsequently building logistic regression for a two class classification. Though this indirect method was able to segregate extreme vs non-extreme *Prakriti*, a direct method would be intuitive, easy to implement and more generalizable. We expect integration of multi-system data with phenotype data will lead to a better resolution for extreme and non-extreme *Prakriti* separation.

It is interesting that stratification within normal subjects can arise from data of multiple levels. These strata could be reflective of a mixture of distinct genetic, and physiological backgrounds and age groups. Though we have assessed the performance of extreme *Prakriti* models in another population, to improve outcome and applicability this needs to be extended to more populations and in this regard a universal predictive model would be an appropriate thing for wider application.

We might have missed out on some of the clinically relevant features due to technical limitation of the modelling methods where missing feature values lead to their omission during model building exercise. Also, rare sub phenotypes associated with particular *Prakriti* although clinically important, might not have been captured. Consideration of differential weightage of questions for estimation of different *Prakriti*, in different age groups, socio-cultural and geo-climatic conditions (ethnicity) might be required in future modelling studies for universal applicability.

## Conclusion

This study thus reveals that the clinical methods of *Prakriti* evaluation are non-empirical and further it can be recapitulated and formalised through advanced machine learning approaches.

Amidst the spectrum of phenotypic heterogeneity even within the genetically homogeneous population, the interconnectedness of sub-phenotypes of different systems get highlighted in the *Prakriti* groups.

Through supervised modelling, we have achieved reduction in features and questions required for accurate *Prakriti* prediction. This would aid the decision making process of *Prakriti* evaluation even by trained Ayurveda physicians. These models would be useful for identification of endo-phenotypes within and across heterogeneous populations and help decipher novel link of genotypes to multisystem phenotypes in association studies.

## Supporting information

S1 Fig Flow-chart depicting the steps of the analysis followed. (PDF)

S2 Fig PCA plot of study population along with other Indian population. (PDF)

S3 Fig MDS visualization of non-extreme *Prakriti* samples with respect to extreme *Prakriti* sample. (PDF)

S4 Fig MDS plot of unsupervised clustering using random forests performed on extreme *Prakriti* male (4a) and female (4b) subjects separately.
(PDF)

S5 Fig Savannah plot for unsupervised clustering of extreme *Prakriti* male and female data. (PDF)

S6 Fig Plot for 39 important features selected from LASSO model. (PDF)

S7 Fig Plot for 61 important features selected from Elastic net model. (PDF)

S8 Fig Plot for 59 important features selected from Random forests model. (PDF)

S1 Table Important Features from LASSO, Elastic net and random forests. (DOCX)

S2 Table Confusion matrices for 10% validation data (Vadu population). (DOCX)

S3 Table Confusion matrices for validation on North Indian population. (DOCX)

S4 Table Confusion matrices for extreme vs non-extreme modelling. (DOCX)

S1 Appendix Modelling of North Indian population data and validation on Vadu cohort data. (DOCX)
