# Supplementary figures and images for: Recapitulation of Ayurveda constitution types by machine learning of phenotypic traits
Source: PLoS One. 2017 Oct 5;12(10):e0185380. doi: 10.1371/journal.pone.0185380 (PMC5628820; doi:10.1371/journal.pone.0185380)

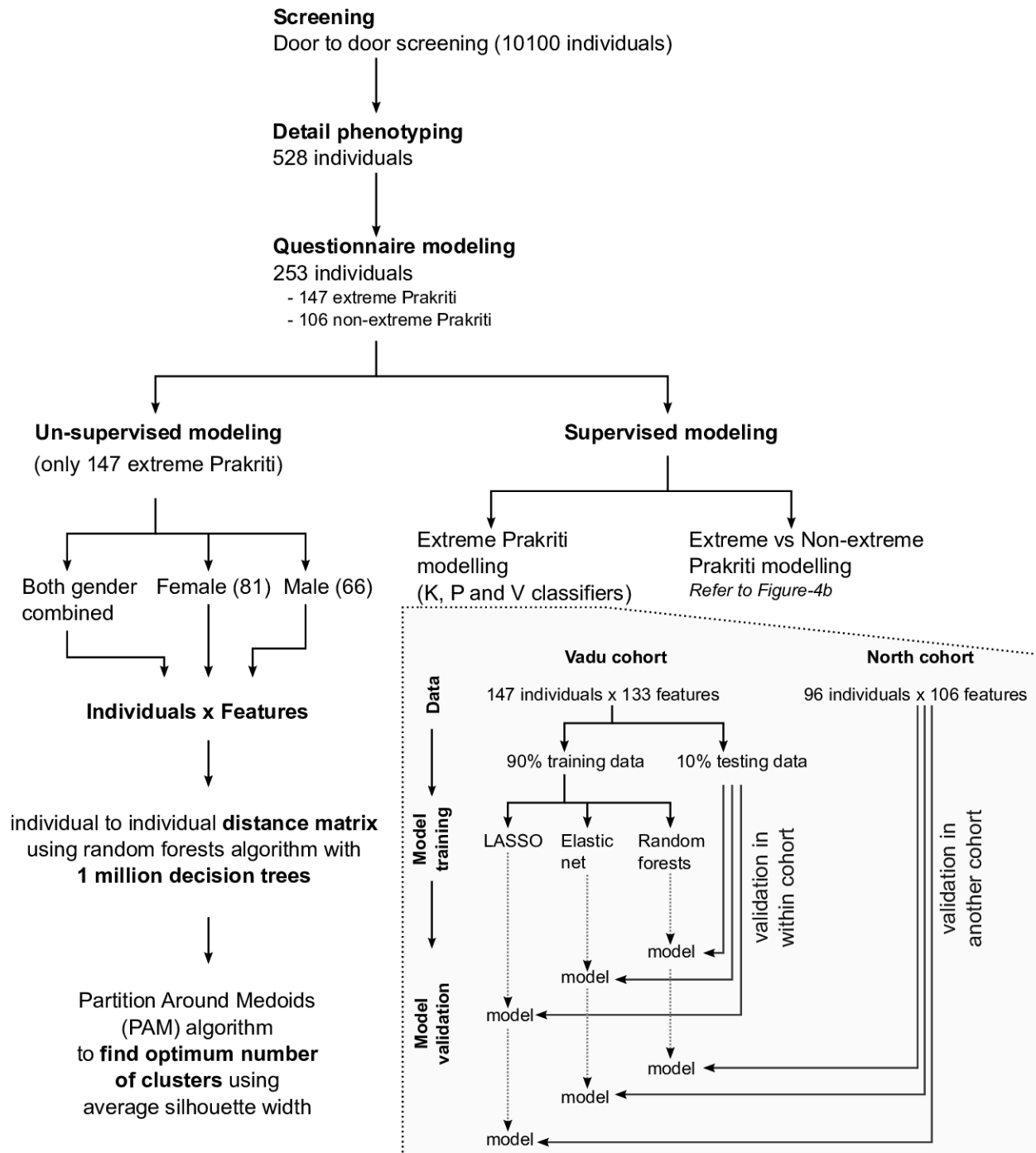

**Supplementary figure S1: Flow chart depicting all the steps employed in the manuscript.**

Supplement: S1 Fig — (PDF) [file pone.0185380.s001.pdf]
