# Supplementary material for: Recapitulation of Ayurveda constitution types by machine learning of phenotypic traits
Source: PLoS One. 2017 Oct 5;12(10):e0185380. doi: 10.1371/journal.pone.0185380 (PMC5628820; doi:10.1371/journal.pone.0185380)
Supplement: S2 Fig — (PDF) [file pone.0185380.s002.pdf]

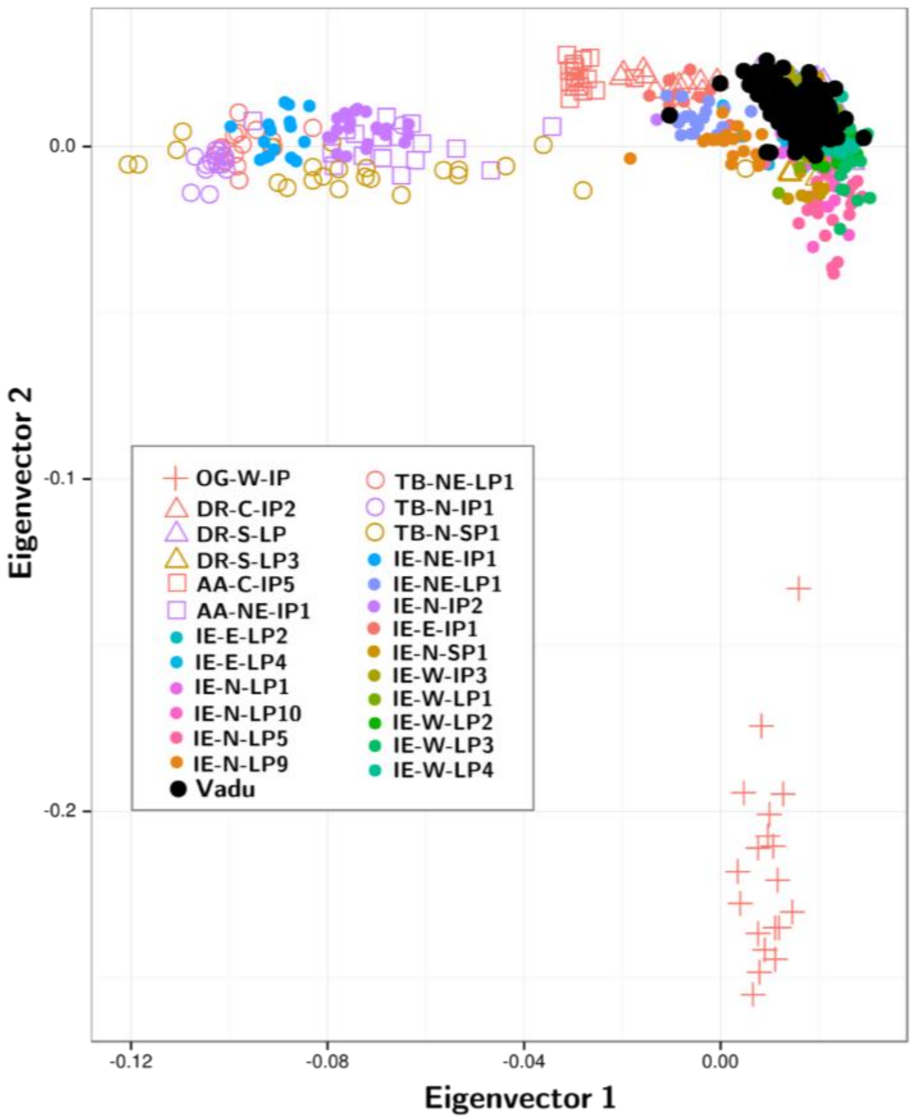

S2 Fig: PCA plot of study (Vadu) population along with other Indian Population: Study population shown in black clusters with other western Indian Population (IE-W-LP1, IE-W-LP2 etc.). This confirms the genetic homogeneity of the study population. The populations naming convention is on the basis of the Indian Genome Variation Consortium panel. (Hum Genet. 2005;118(1):1-11.) The populations are coded on the basis of linguistic lineage (AA, AustroAsiatic; IE, Indo-European; DR, Dravidian; and TB, Tibeto-Burman) followed by geographical location (N, north; NE, northeast; W, west; E, east; S, south; and C, central) and ethnic category (LP, castes; Sp, religious groups; and IP, tribes)
