# Supplementary material for: Recapitulation of Ayurveda constitution types by machine learning of phenotypic traits
Source: PLoS One. 2017 Oct 5;12(10):e0185380. doi: 10.1371/journal.pone.0185380 (PMC5628820; doi:10.1371/journal.pone.0185380)
Supplement: S3 Fig — (PDF) [file pone.0185380.s003.pdf]

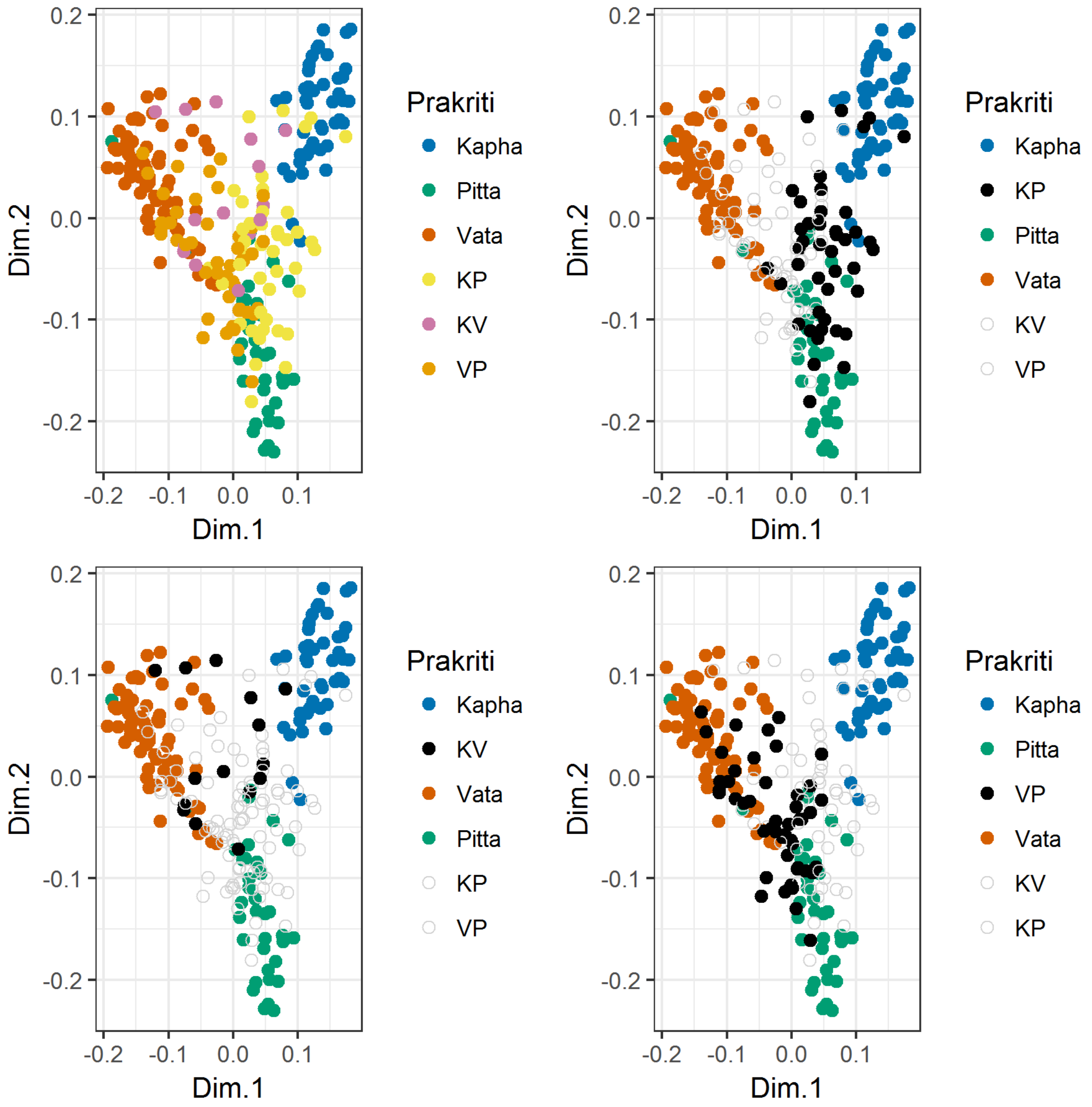

Supplementary Figure S3: MDS visualization of non-extreme Prakriti with respect to Extreme Prakriti samples: Top left figure represent the plot for extreme and non-extreme Prakriti samples together, for better visualization this figure has been split into three other figures (top right and bottom panel) where only one non-extreme Prakriti has been visualized at a time and other two non-extreme Prakriti has been made lighter in appearance. Non-extreme *Prakriti* types were found to be intermediate of their respective extreme type e.g. KP *Prakriti* type (top right) occupies intermediate space of *Kapha* and *Pitta* extreme *Prakriti*. A similar trend can be seen in case of other non-extreme *Prakriti* types (bottom panel).
