# Supplementary material for: Recapitulation of Ayurveda constitution types by machine learning of phenotypic traits
Source: PLoS One. 2017 Oct 5;12(10):e0185380. doi: 10.1371/journal.pone.0185380 (PMC5628820; doi:10.1371/journal.pone.0185380)
Supplement: S4 Fig — MDS plot of unsupervised clustering using random forests performed on extreme Prakriti male (4a) and female (4b) subjects separately. (PDF) [file pone.0185380.s004.pdf]

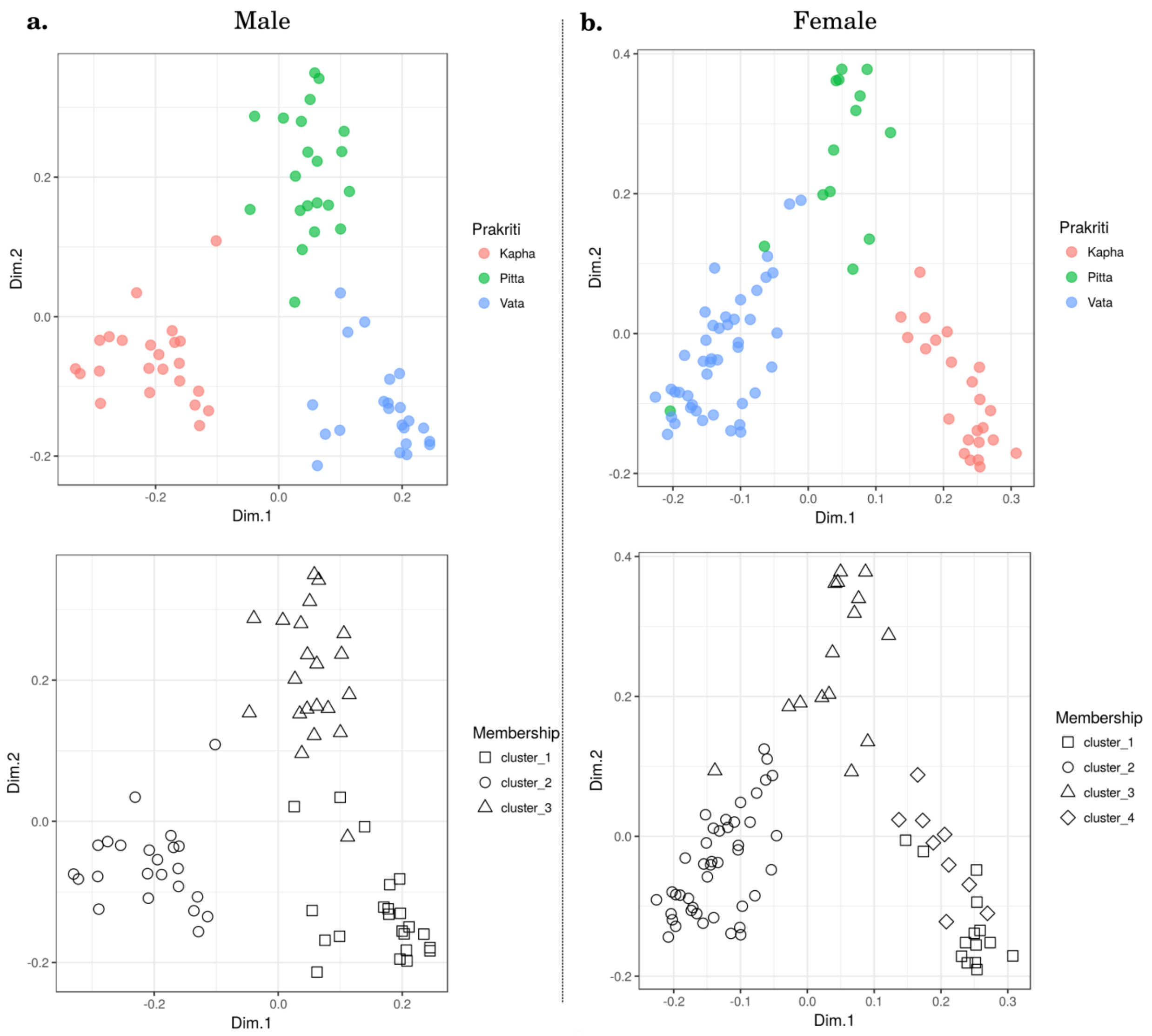

Supplementary figures S4: MDS plot of unsupervised clustering using random forests performed on male (4a) and female (4b) extreme *Prakriti* subjects separately. In the case of male subjects three clusters were found (bottom panel, left) while in case of female samples four clusters were found (bottom panel, right).
