# Supplementary material for: Recapitulation of Ayurveda constitution types by machine learning of phenotypic traits
Source: PLoS One. 2017 Oct 5;12(10):e0185380. doi: 10.1371/journal.pone.0185380 (PMC5628820; doi:10.1371/journal.pone.0185380)
Supplement: S5 Fig — (PDF) [file pone.0185380.s005.pdf]

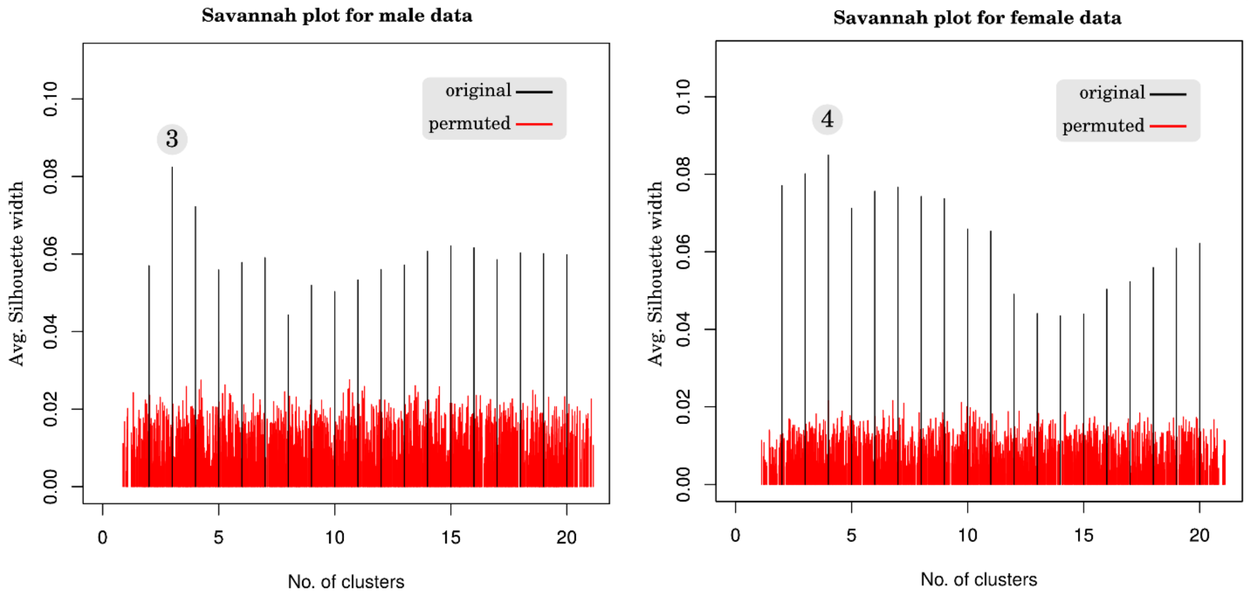

Supplementary figures S5: Savannah plot for unsupervised clustering of extreme Prakriti male and female samples separately. Like in case of combined data black line represents silhouette width for original data which indicates three and four clusters for male and female respectively. Permuted silhouette (red lines) width are smaller in size and indicates robust clusters.
