# Supplementary material for: Recapitulation of Ayurveda constitution types by machine learning of phenotypic traits
Source: PLoS One. 2017 Oct 5;12(10):e0185380. doi: 10.1371/journal.pone.0185380 (PMC5628820; doi:10.1371/journal.pone.0185380)
Supplement: S6 Fig — (PDF) [file pone.0185380.s006.pdf]

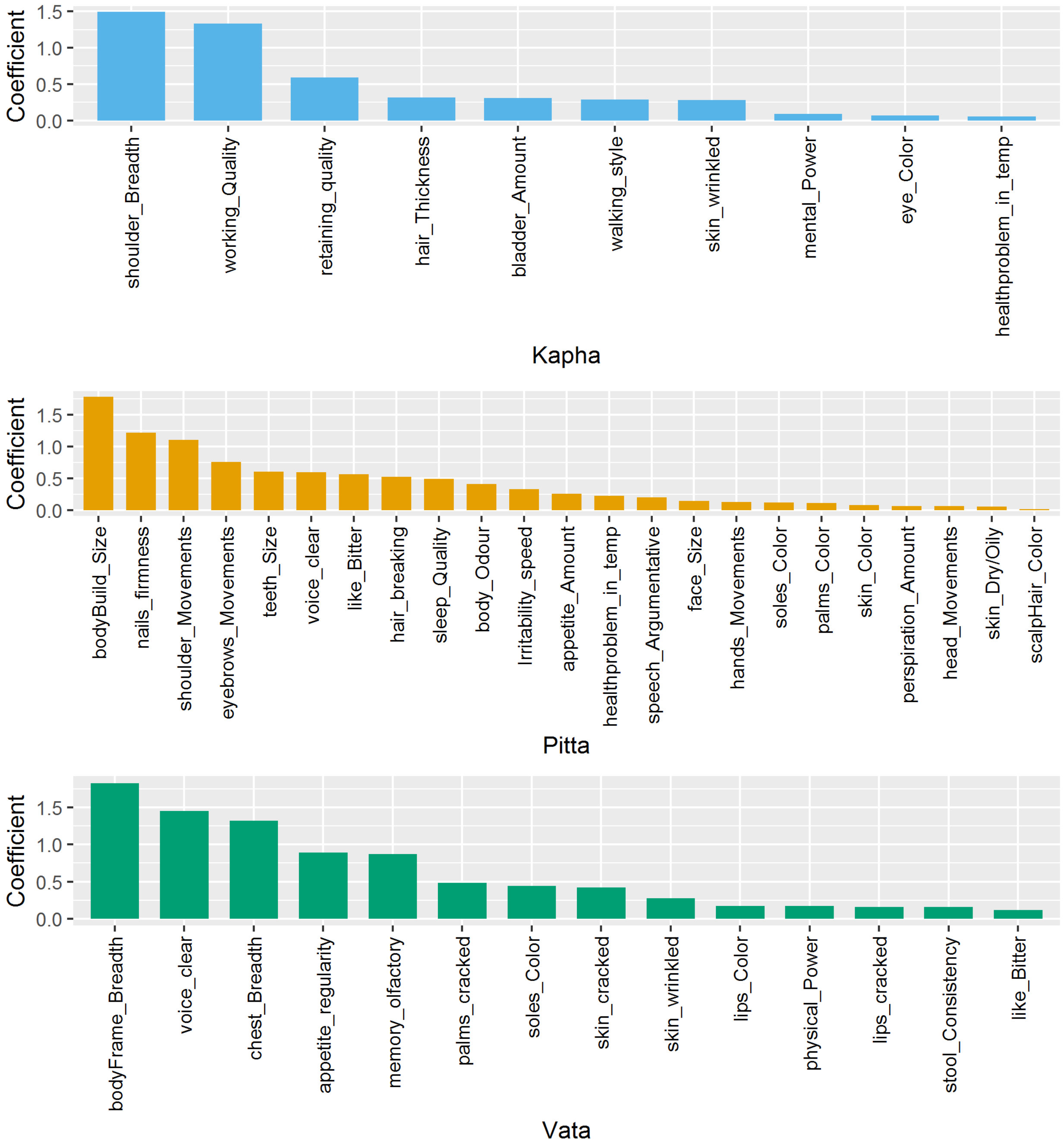

Figure S6: Important variable plot for 39 variables from LASSO model: Y-axis represents the absolute value of the coefficients for the features selected from the lasso model.
