# Supplementary material for: Recapitulation of Ayurveda constitution types by machine learning of phenotypic traits
Source: PLoS One. 2017 Oct 5;12(10):e0185380. doi: 10.1371/journal.pone.0185380 (PMC5628820; doi:10.1371/journal.pone.0185380)
Supplement: S7 Fig — (PDF) [file pone.0185380.s007.pdf]

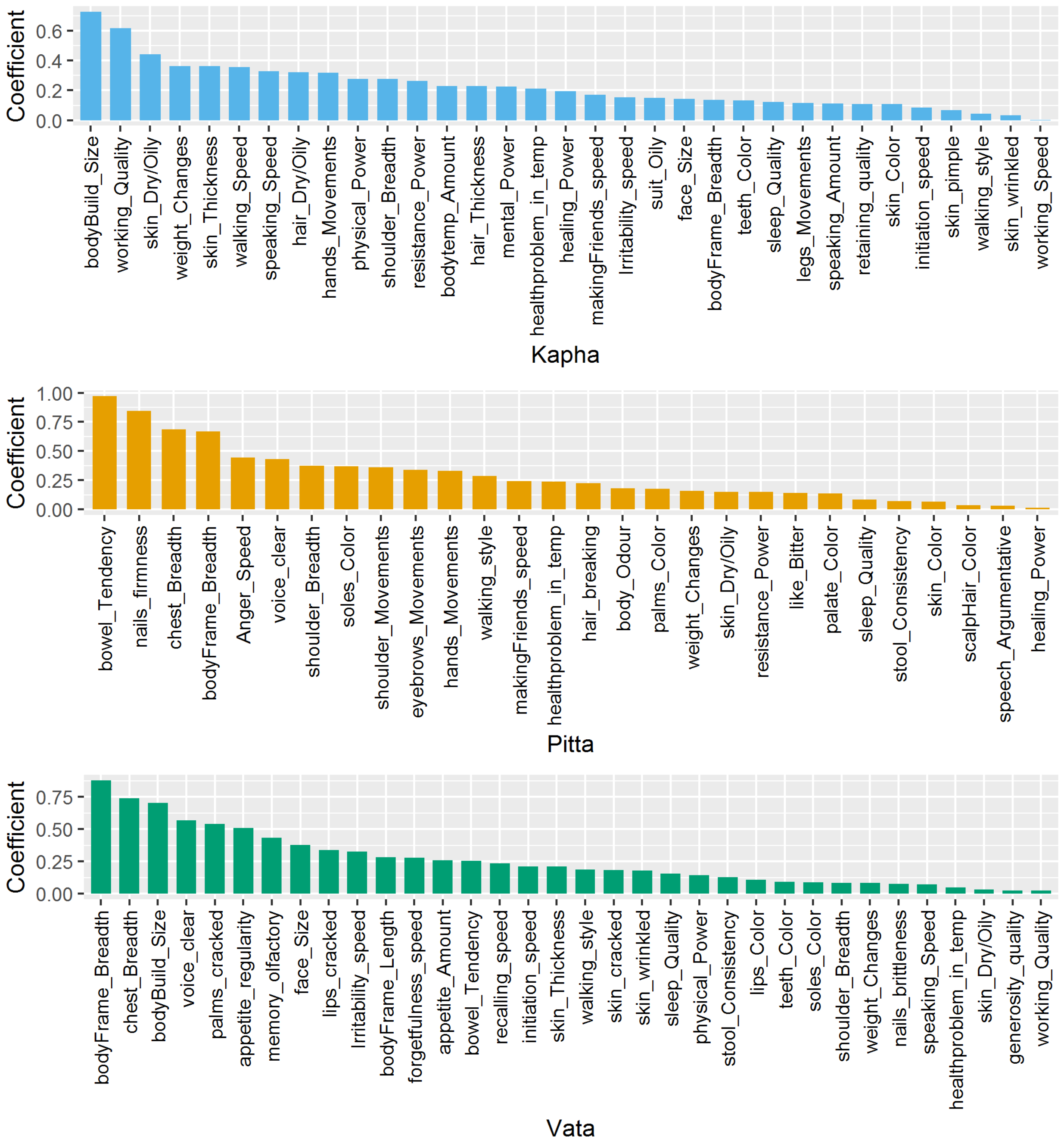

Figure S7: **Important variable plot for 61 variables from elastic net model**: Plot represents *Prakriti* wise important variables. Y-axis represents the absolute value of the coefficient for the features selected from elastic net model. These features are superset of features selected from LASSO model.
