# Supplementary material for: Recapitulation of Ayurveda constitution types by machine learning of phenotypic traits
Source: PLoS One. 2017 Oct 5;12(10):e0185380. doi: 10.1371/journal.pone.0185380 (PMC5628820; doi:10.1371/journal.pone.0185380)
Supplement: S8 Fig — (PDF) [file pone.0185380.s008.pdf]

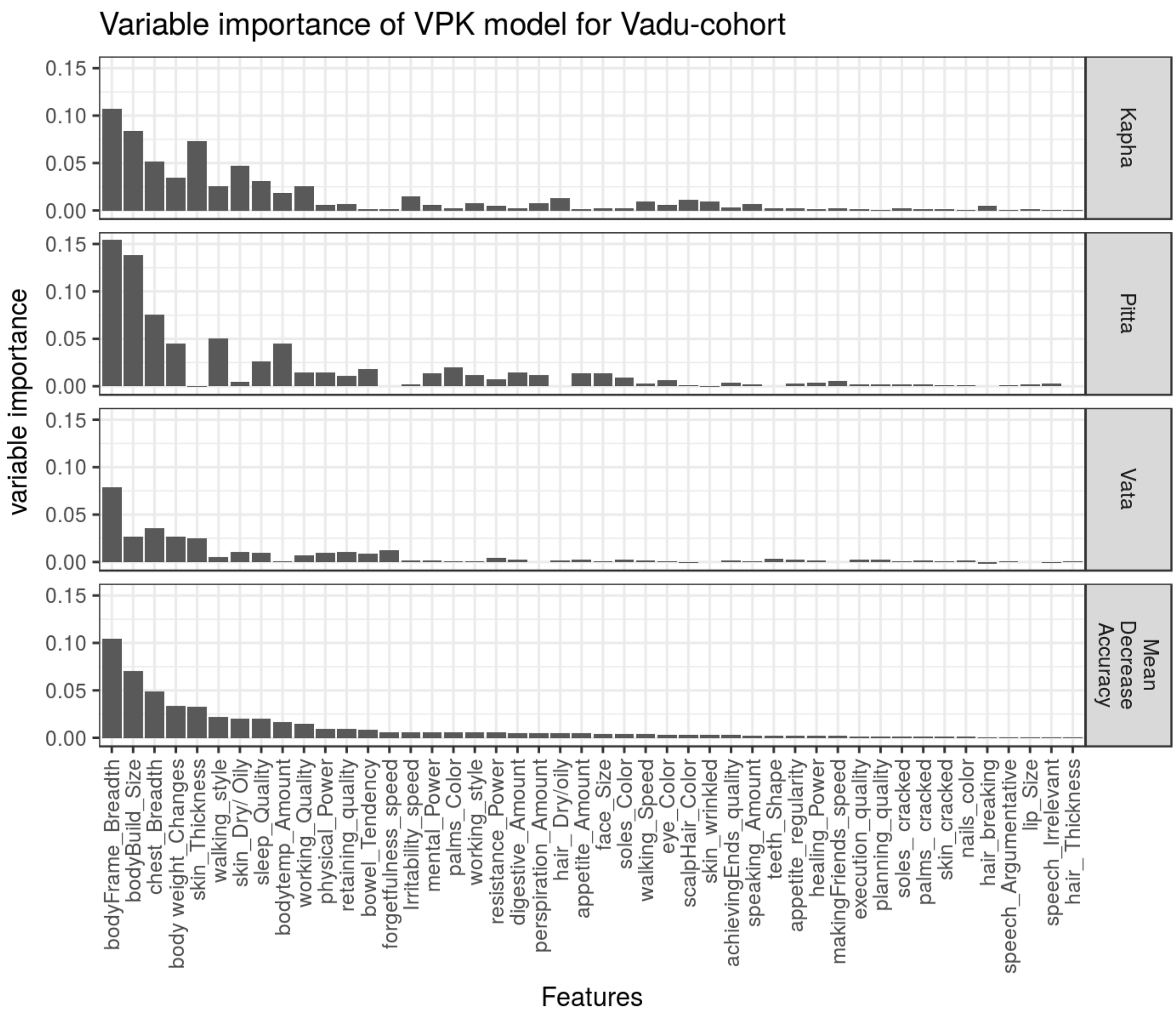

Figure S8: **Important variable plot for 59 variables from random forests model**: Random forests algorithm provides variable importance for overall model as well as for class-wise. Variables with large mean decrease accuracy represents more importance to discriminate among *Prakriti* classes.
