# Supplementary material for: Recapitulation of Ayurveda constitution types by machine learning of phenotypic traits
Source: PLoS One. 2017 Oct 5;12(10):e0185380. doi: 10.1371/journal.pone.0185380 (PMC5628820; doi:10.1371/journal.pone.0185380)
Supplement: S1 Table — (DOCX) [file pone.0185380.s009.docx]

**Table S1: Important features from LASSO, Elastic-net and Random forests:** 39, 61 and 59 features were important from the LASSO, Elastic net and Random forests modelling of Vadu cohort data. 31 features were common between all of three and represented in blue colour in table (top 31 values). While features obtained through LASSO were subset of Elastic net features, some features were unique to Elastic net and Random Forests.
