# Supplementary material for: Recapitulation of Ayurveda constitution types by machine learning of phenotypic traits
Source: PLoS One. 2017 Oct 5;12(10):e0185380. doi: 10.1371/journal.pone.0185380 (PMC5628820; doi:10.1371/journal.pone.0185380)
Supplement: S2 Table — (DOCX) [file pone.0185380.s010.docx]

**2a. LASSO**

|  | | **REFERENCE** | | |
| --- | --- | --- | --- | --- |
|  |  | **Kapha** | **Pitta** | **Vata** |
| **PREDICTED** | **Kapha** | 5 | 0 | 0 |
|  | **Pitta** | 0 | 4 | 0 |
|  | **Vata** | 0 | 0 | 7 |

**2b. Elastic net**

|  |  | **REFERENCE** | | |
| --- | --- | --- | --- | --- |
|  |  | **Kapha** | **Pitta** | **Vata** |
| **PREDICTED** | **Kapha** | 5 | 0 | 0 |
|  | **Pitta** | 0 | 4 | 0 |
|  | **Vata** | 0 | 0 | 7 |

**2c. Random forests**

|  |  | **REFERENCE** | | |
| --- | --- | --- | --- | --- |
|  |  | **Kapha** | **Pitta** | **Vata** |
| **PREDICTED** | **Kapha** | 5 | 0 | 0 |
|  | **Pitta** | 0 | 4 | 0 |
|  | **Vata** | 0 | 0 | 7 |

**Table S2: Confusion matrices for 10% validation data (Vadu population):** All the three methods performed equally well on 10% left –out set from Vadu population. Columns in the table represent no of individual present in validation set while rows presents predicted no of individuals in a class (2a, 2b and 2c).
