# Supplementary material for: Recapitulation of Ayurveda constitution types by machine learning of phenotypic traits
Source: PLoS One. 2017 Oct 5;12(10):e0185380. doi: 10.1371/journal.pone.0185380 (PMC5628820; doi:10.1371/journal.pone.0185380)
Supplement: S3 Table — (DOCX) [file pone.0185380.s011.docx]

**3a. LASSO**

|  |  | **REFERENCE** | | |
| --- | --- | --- | --- | --- |
|  |  | **Kapha** | **Pitta** | **Vata** |
| **PREDICTED** | **Kapha** | 27 | 0 | 0 |
|  | **Pitta** | 2 | 24 | 2 |
|  | **Vata** | 0 | 5 | 36 |

**3b. Elastic net**

|  |  | **REFERENCE** | | |
| --- | --- | --- | --- | --- |
|  |  | **Kapha** | **Pitta** | **Vata** |
| **PREDICTED** | **Kapha** | 28 | 0 | 0 |
|  | **Pitta** | 1 | 25 | 1 |
|  | **Vata** | 0 | 4 | 37 |

**3c. Random forests**

|  |  | **REFERENCE** | | |
| --- | --- | --- | --- | --- |
|  |  | **Kapha** | **Pitta** | **Vata** |
| **PREDICTED** | **Kapha** | 29 | 1 | 0 |
|  | **Pitta** | 0 | 23 | 1 |
|  | **Vata** | 0 | 5 | 37 |

**Table S3: Confusion matrices for validation on North India Population** : Upper panel shows predicted and original no of individual in each class generated from three methods LASSO (3a), elastic net (3b) and random forests (3c) respectively.
