# Supplementary material for: Recapitulation of Ayurveda constitution types by machine learning of phenotypic traits
Source: PLoS One. 2017 Oct 5;12(10):e0185380. doi: 10.1371/journal.pone.0185380 (PMC5628820; doi:10.1371/journal.pone.0185380)
Supplement: S4 Table — (DOCX) [file pone.0185380.s012.docx]

**4a. LASSO**

|  |  | **REFERENCE** | |
| --- | --- | --- | --- |
|  |  | **Extreme** | **Non extreme** |
| **PREDICTED** | **Extreme** | 15 | 0 |
|  | **Non extreme** | 2 | 9 |

**4b. Elastic net**

|  |  | **REFERENCE** | |
| --- | --- | --- | --- |
|  |  | **Extreme** | **Non**  **extreme** |
| **PREDICTED** | **Extreme** | 14 | 1 |
|  | **Non**  **extreme** | 0 | 11 |

**4c. Random forests**

|  |  | **REFERENCE** | |
| --- | --- | --- | --- |
|  |  | **Extreme** | **Non**  **extreme** |
| **PREDICTED** | **Extreme** | 14 | 1 |
|  | **Non**  **extreme** | 1 | 10 |

**Table S4: Confusion matrices for extreme vs non-extreme modelling:** Columns of these classification tables (4a, 4b and 4c) represents original number of samples in each class. Rows represents number of predicted samples in each class.
