# Supplementary material for: Recapitulation of Ayurveda constitution types by machine learning of phenotypic traits
Source: PLoS One. 2017 Oct 5;12(10):e0185380. doi: 10.1371/journal.pone.0185380 (PMC5628820; doi:10.1371/journal.pone.0185380)
Supplement: S1 Appendix — (DOCX) [file pone.0185380.s013.docx]

**S1 Appendix:**

**Modelling of North Indian population data and validation on Vadu cohort data**

**Modelling of North India data:**

We have also built models on North India extreme *Prakriti* data. The same approach of data splitting into 90% training and 10% testing data was adopted to build models .The models built were validated on Vadu data

**Result:**

All the three algorithms were able to classify samples from Vadu population with considerable accuracy. However, accuracy was bit low in case of classification of Vata samples (65%) using lasso model (Supplementary Table S4).

**Supplementary Table S4:**

1. **LASSO**

|  | | **REFERENCE** | | |
| --- | --- | --- | --- | --- |
|  |  | **Kapha** | **Pitta** | **Vata** |
| **PREDICTED** | **Kapha** | 40 | 1 | 2 |
|  | **Pitta** | 6 | 33 | 21 |
|  | **Vata** | 0 | 1 | 43 |

1. **Elastic net**

|  | | **REFERENCE** | | |
| --- | --- | --- | --- | --- |
|  |  | **Kapha** | **Pitta** | **Vata** |
| **PREDICTED** | **Kapha** | 38 | 1 | 0 |
|  | **Pitta** | 8 | 33 | 12 |
|  | **Vata** | 0 | 1 | 54 |

1. **Random forests**

|  | | **REFERENCE** | | |
| --- | --- | --- | --- | --- |
|  |  | **Kapha** | **Pitta** | **Vata** |
| **PREDICTED** | **Kapha** | 43 | 1 | 0 |
|  | **Pitta** | 3 | 33 | 8 |
|  | **Vata** | 0 | 1 | 58 |

1. **Model summary**

|  | **Sensitivity (%)** | | | **Specificity (%)** | | |
| --- | --- | --- | --- | --- | --- | --- |
|  | **LASSO** | **Elastic**  **net** | **Random forests** | **LASSO** | **Elastic**  **net** | **Random forests** |
| **Kapha** | 86.96 | 82.60 | 93.48 | 97.03 | 99.00 | 99.01 |
| **Pitta** | 94.29 | 94.29 | 94.29 | 75.89 | 82.14 | 90.18 |
| **Vata** | 65.15 | 81.81 | 87.88 | 98.77 | 98.76 | 98.77 |
